# Uncovering Bioactive Compounds in Propolis Extracts Prior to Isolation Through NMR Chemometric Analysis

**DOI:** 10.3390/molecules31101742

**Published:** 2026-05-20

**Authors:** Maria-Ioanna Stavropoulou, Antigoni Cheilari, Konstantia Graikou, Ioanna Chinou, Nektarios Aligiannis

**Affiliations:** Laboratory of Pharmacognosy and Natural Products Chemistry, Faculty of Pharmacy, National and Kapodistrian University of Athens, Panepistimiopolis Zografou, 15771 Athens, Greecekgraikou@pharm.uoa.gr (K.G.); ichinou@pharm.uoa.gr (I.C.); aligiannis@pharm.uoa.gr (N.A.)

**Keywords:** Greek-propolis, NMR-HeteroCovariance approach, metabolomics, flavonoids, diterpenes, collagenase inhibition, DPPH assay

## Abstract

Propolis is a resinous bee product with a long history of medicinal use, valued for its antimicrobial, antioxidant and anti-inflammatory properties. Identification of its key bioactive constituents would enable chemical standardization and quality control of propolis-based products, once their activity is confirmed. In the current study we investigated a Greek propolis sample (PR09) belonging to a phenolic-rich type, dominated by flavonoids. After extraction and fractionation with Fast Centrifugal Chromatography (FCPC), the fractions were evaluated for their DPPH and collagenase inhibitory activity while their NMR metabolic profiles were recorded. NMR HeteroCovariance Approach (NMR-HetCA) analysis of PR09 propolis methanolic extract revealed the presence of 26 secondary metabolites: seven diterpenes, 13 flavonoids and six caffeic acid esters. All compounds were identified from NMR-HetCA and Statistical Total Correlation Spectroscopy (STOCSY) plots prior to their isolation. NMR-HetCA analysis indicated that caffeic acid derivatives were the most potent inhibitors of the DPPH free radical and collagenase. Additionally, galangin (**11**) and 3-O-methyl galangin (**24**) appeared to contribute considerably to the antioxidant activity, while together with pinocembrin (**12**), they all contributed to the extract’s collagenase inhibitory activity. In contrast, metabolites such as isocupressic acid (**8**), 13-*epi*-cupressic acid (**18**), pinostrobin (**17**) and chrysin (**7**) appeared not to contribute to the observed activities. Bioassays of selected metabolites confirmed the NMR-HetCA’s predictions, with caffeic acid phenethyl ether (**1**) exhibiting very high inhibition (92.54 ± 0.16%), and notable collagenase inhibition close to 50% (at 100 μg/mL). Overall, the findings demonstrate that NMR-HetCA enables rapid identification of bioactive compounds in propolis extracts and is proposed as a tool in accelerating the evaluation of propolis samples prior to laborious isolation procedures.

## 1. Introduction

Propolis is a complex resinous bee product collected by honeybees from various buds and exudates, which is then enzymatically transformed and used by the bees, mainly for the protection of their hives against intruders [1,2]. It has been used as a remedy since antiquity, recognized for its diverse pharmacological activities [3]. Its medicinal properties were documented by Roman and Greek physicians, including Aristoteles, Dioscorides, Pliny and Galen, while the Egyptians employed propolis for wound healing and the embalming of corpses (preservation from decomposition) [4,5]. Nowadays, propolis is still used in folk medicine to treat open wounds, burns and other skin conditions such as eczema and psoriasis [6]. Moreover, it has found modern applications as a natural food additive, as a cosmeceutical agent and/or as a functional food ingredient [7].

The chemical composition of propolis is highly variable and influenced by several factors, including the botanical sources around the hive, the seasonal variations, the climatic conditions and the species of bees. As a result, numerous distinct propolis types have been identified around the world. Greece, with its diverse geomorphology and climate, hosts rich biodiversity that influences the chemical variability of local propolis. In our previous study [8], methanolic extracts of Greek propolis were analyzed using High-Performance Thin Layer Chromatography (HPTLC) and Nuclear Magnetic Resonance (NMR), revealing significant chemical diversity. Based on multivariate statistical analysis, we identified three major groups: (a) Group I, consisting of samples rich in terpenoids, which presented low antioxidant but high anti-tyrosinase activity; (b) Group II, consisting of samples rich in flavonoids that showed the highest antioxidant and anti-collagenase activities; and (c) Group III, consisting of samples with lower flavonoid content than the samples of Group II, which exhibited moderate antioxidant, anti-collagenase and anti-tyrosinase activities.

Given the potent antioxidant and collagenase inhibitory activities observed in Group II, activities linked to wound-healing and anti-aging effects, sample PR09 (from Mt. Olympus) was selected for further investigation. This study aims to rapidly identify specific secondary metabolites in PR09 with antioxidant and/or anti-collagenase activity prior to their isolation. Being aware of the complexity of the extract, as indicated by spectral data, NMR HeteroCovariance Approach (NMR-HetCA) was employed to identify the relevant compounds responsible for the activities of the extract. NMR-HetCA is a methodology used by our group to detect the bioactive components of a mixture prior to their isolation. NMR-HetCA correlates ^1^H-NMR spectral features with bioactivity data, generating pseudo-spectra (HetCA plots) in which covariance and correlation coefficients highlight signals associated with biological effects. NMR-HetCA was recently evaluated in depth with a success rate of 63.2% in identifying bioactive substances prior to isolation [9]. Fast Centrifugal Partition Chromatography (FCPC) is a liquid–liquid chromatographic technique relying exclusively on the partition of compounds between two immiscible liquid phases. The absence of a solid stationary phase eliminates irreversible adsorption, allowing full sample recovery—including the option to retrieve the entire injected material if fractionation needs to be repeated. FCPC naturally generates a gradient of compound concentrations across fractions, a prerequisite for the NMR-HetCA bioactivity correlation approach. Additional advantages include reduced solvent consumption during elution and the availability of a broad range of biphasic solvent systems spanning a wide polarity range, enabling tailored fractionation for chemically diverse extracts. In the present study, System Q of the ARIZONA family was selected, providing effective separation of the phenolic-rich PR09 extract [10].

Collagenase (matrix metalloproteinase-1, MMP-1) plays a central role in extracellular matrix degradation, and its inhibition is directly relevant to the wound-healing and skin-protective properties attributed to propolis in traditional and contemporary use. Several studies have demonstrated that propolis extracts and compounds—particularly phenolic acids and flavonoids—inhibit MMP activity in keratinocyte and fibroblast models, supporting the mechanistic basis for their topical application in wound care and dermatological preparations [11,12,13,14,15]. Collagenase inhibition, thus, represents a biologically meaningful endpoint connecting the chemical composition of propolis to its documented therapeutic applications. While DPPH radical scavenging lacks direct in vivo physiological relevance [16], it is retained here as a well-established chemical property assay to demonstrate the capacity of NMR-HetCA to simultaneously correlate spectral profiles with multiple and distinct bioactivity endpoints.

The application of NMR-HetCA to propolis extracts aims not only to chemically characterize PR09, but also to provide a more efficient alternative to traditional bioactivity-guided fractionation. Conventional methods are often time-consuming and costly, requiring weeks or months to isolate and characterize bioactive constituents. They also frequently lead to isolation of moderately active compounds or rediscovery of already known natural products, limiting efficiency in the discovery of novel bioactives. Moreover, the identification of specific bioactive constituents through NMR-HetCA creates a direct pathway toward chemical standardization of propolis-based products. Once a putative active compound is identified, its role can be validated later in established in vivo models of wound healing or skin inflammation. Moreover, this approach can be utilized for future standardization of propolis products with defined concentrations of the identified active constituents, thereby establishing a concentration–activity relationship suitable for quality control purposes. The present study, by rapidly identifying and ranking the bioactive compounds within a complex propolis extract, is intended to support and accelerate this pipeline.

In conclusion, this study contributes to the characterization and validation of Greek propolis by integrating advanced NMR-based metabolic profiling with activity correlation techniques. Our findings accelerate the discovery of bioactive compounds with potential therapeutic relevance and enhance understanding of the chemical basis behind the traditional use of propolis in skin-related ailments.

## 2. Results

### 2.1. Extraction of Propolis and Fractionation by Fast Centrifugal Partition Chromatography (FCPC)

The studied propolis sample (PR09) was collected from the region of Mount Olympus, northern Greece. Defatting with n-heptane (2 × 20 mL) was followed by methanolic extraction (2 × 20 mL) in an ultrasonic bath, yielding 1.27 g of dry extract (31.72%). Fractionation by FCPC using the Arizona Q [10] solvent system (heptane/ethyl acetate/methanol/water, 3:2:3:2) yielded 59 fractions with adequate variation in compound concentrations across the fractionation gradient, serving NMR-HetCA prerequisites.

### 2.2. HPTLC Chemical Profiling of FCPC Fractions

**Figure 1:**
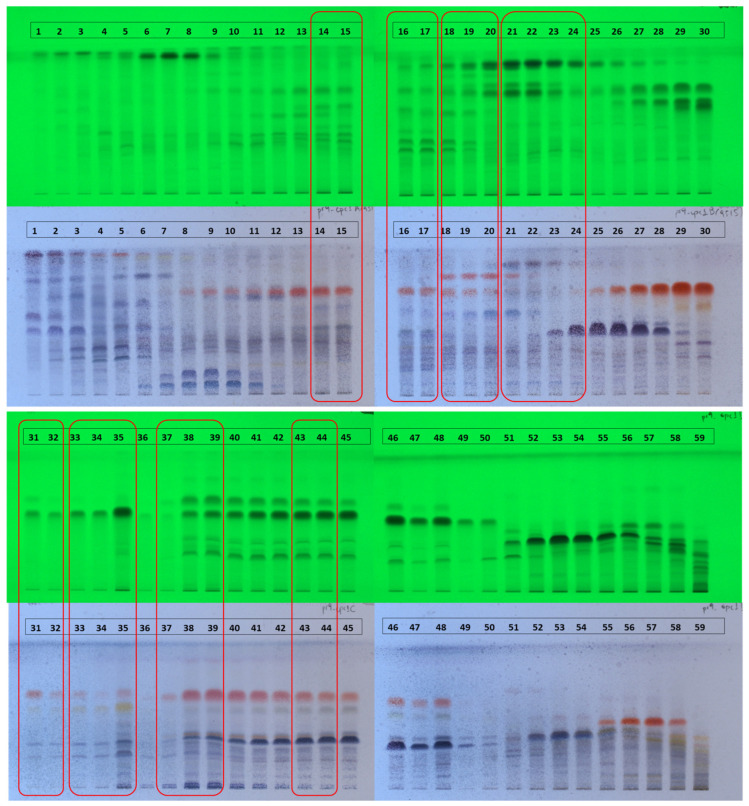
HPTLC profiling of all 59 FCPC fractions before merging on normal-phase silica gel plates, developed with dichloromethane:methanol (90:10). Documentation was performed under UV light at 254 nm (**top**), revealing flavonoids and phenolic acid derivatives by absorbance, and under white light after derivatization with sulfuric vanillin reagent followed by heating (**bottom**), enabling detection of terpenes as blue-purple spots and flavonoids as yellow-orange spots. Based on the observed chemical profiles and concentration gradients across fractions, the initial 59 fractions were merged into 43 final fractions (indicated by bracketing), balancing chemical diversity with adequate concentration variation required for NMR-HetCA analysis.

HPTLC profiling of the 59 FCPC fractions revealed three main chemical categories (Figure 1): terpenes, detected as blue-purple spots under white light after spraying with sulfuric vanillin and heating; flavonoids, identified by absorbance at 254 nm and as yellow-orange spots post-spraying and heating; and phenolic acid derivatives, identified by absorbance at 254 nm and appearing as purple spots post-spraying and heating, predominantly in fractions 52–54 (Figure S1). Based on the observed distribution and concentration gradients across fractions, the 59 fractions were merged into 43 final fractions as indicated by bracketing in Figure 1.

### 2.3. In Vitro Collagenase Inhibition Assay

**Figure 2:**
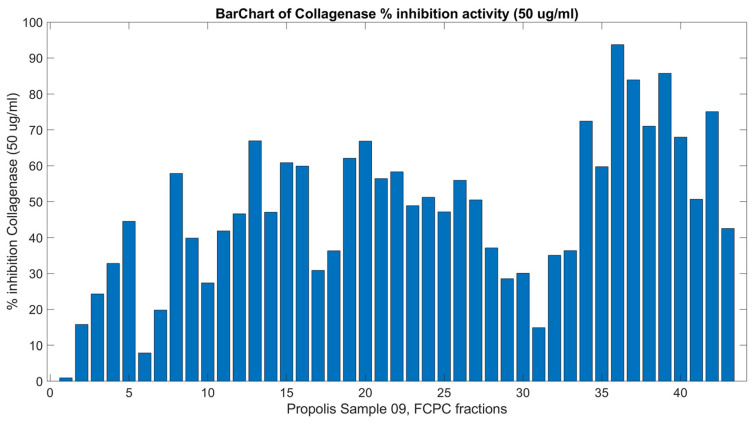
Collagenase-inhibitory activity of all 43 final FCPC fractions of PR09 propolis methanolic extract, at a concentration of 50 μg/mL. Inhibition values were used for correlation with ^1^H NMR spectral data in the NMR-HetCA analysis. Complete numerical values with standard deviations are provided in Table S1.

The crude extract and the FCPC-derived fractions from the methanolic extract of PR09 were evaluated for their in vitro ability to inhibit collagenase. In our previous study [8], the crude methanolic extracts of propolis samples were tested initially at 100 μg/mL, exhibiting a high activity, which is not convenient for the application of NMR-HetCA, since the concentration variance is crucial for the correlation of spectral data and bioactivity. Hence, the crude methanolic extracts of propolis were tested at a final concentration of 50 μg/mL, with PR09 exhibiting 69.79% collagenase inhibition [8]. Evaluations of the FCPC fractions at the same concentration (C = 50 μg/mL) are presented in Figure 2 and Table S1. In more detail, the most potent inhibitory activity was observed from fractions PR09_Fr52–PR09_Fr56 (67.99–93.73%), PR09_Fr13, PR09_Fr18, PR09_Fr29, PR09_Fr30, PR09_Fr50 and PR09_Fr58 (over 60%). Fractions PR09_Fr5, PR09_Fr8, PR09_Fr11, PR09_Fr12, PR09_Fr14, PR09_Fr21, PR09_Fr31-42, PR09_Fr51, PR09_Fr57 and PR09_Fr59 also showed significant activity (40–60%).

### 2.4. DPPH Assay

**Figure 3:**
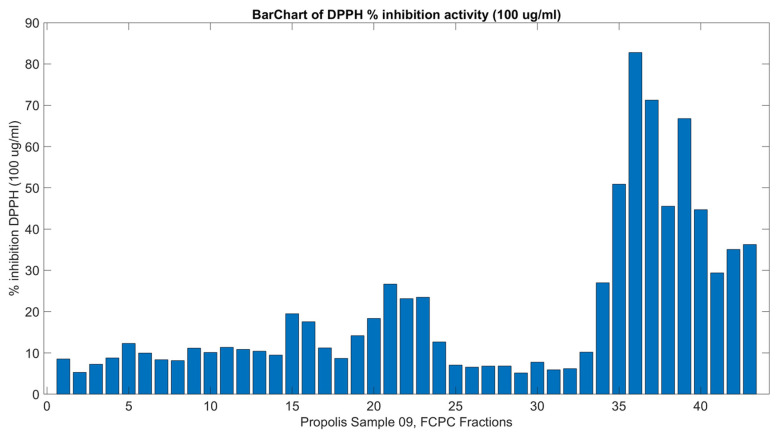
DPPH-inhibitory activity of all 43 final FCPC fractions of PR09 propolis methanolic extract, at a concentration of 100 μg/mL. Inhibition values were used for correlation with ^1^H NMR spectral data in the NMR-HetCA analysis. Complete numerical values with standard deviations are provided in Table S2.

The antioxidant activity of the crude methanolic extract and the FCPC fractions was determined using the DPPH assay. The crude extract exhibited 65.38% inhibition at a final concentration of 100 μg/mL. Among the fractions, the most potent antioxidant activity was observed in PR09_Fr52 and PR09_Fr53 (82.77% and 71.23%, respectively), while fractions PR09_Fr51, PR09_Fr54-PR09_Fr56, PR09_Fr58 and PR09_Fr59 also showed strong activity (35.07–66.8%). The results of the fractions are displayed in Figure 3 and Table S2 as percentages of DPPH inhibition in 100 μg/mL.

### 2.5. NMR Spectroscopy and HeteroCovariance Approach (NMR-HetCA)

**Figure 4:**
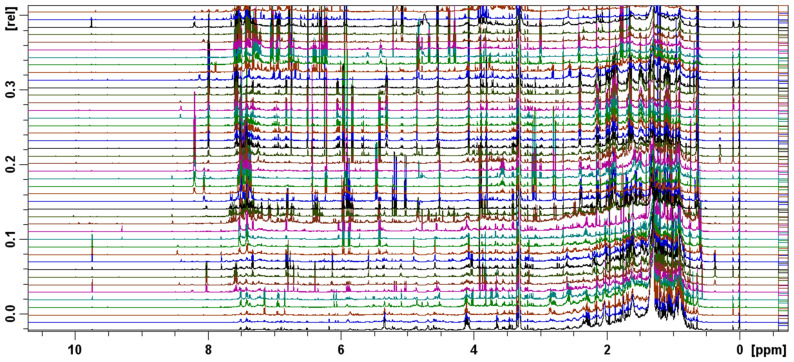
Stacked plot of ^1^H NMR spectra of the 43 final FCPC fractions of PR09 propolis methanolic extract, recorded in methanol-*d*_4_ at 298 K. Spectra are displayed in order of fraction number (bottom to top), illustrating the variation in metabolite composition and relative concentrations across the fractionation gradient. This spectral dataset was used as input for NMR-HetCA and STOCSY analyses.

NMR metabolic profiling of the FCPC fractions revealed distinct chemical patterns across the samples, as illustrated in Figure 4 and in detail in Figure S2.

**Figure 5:**
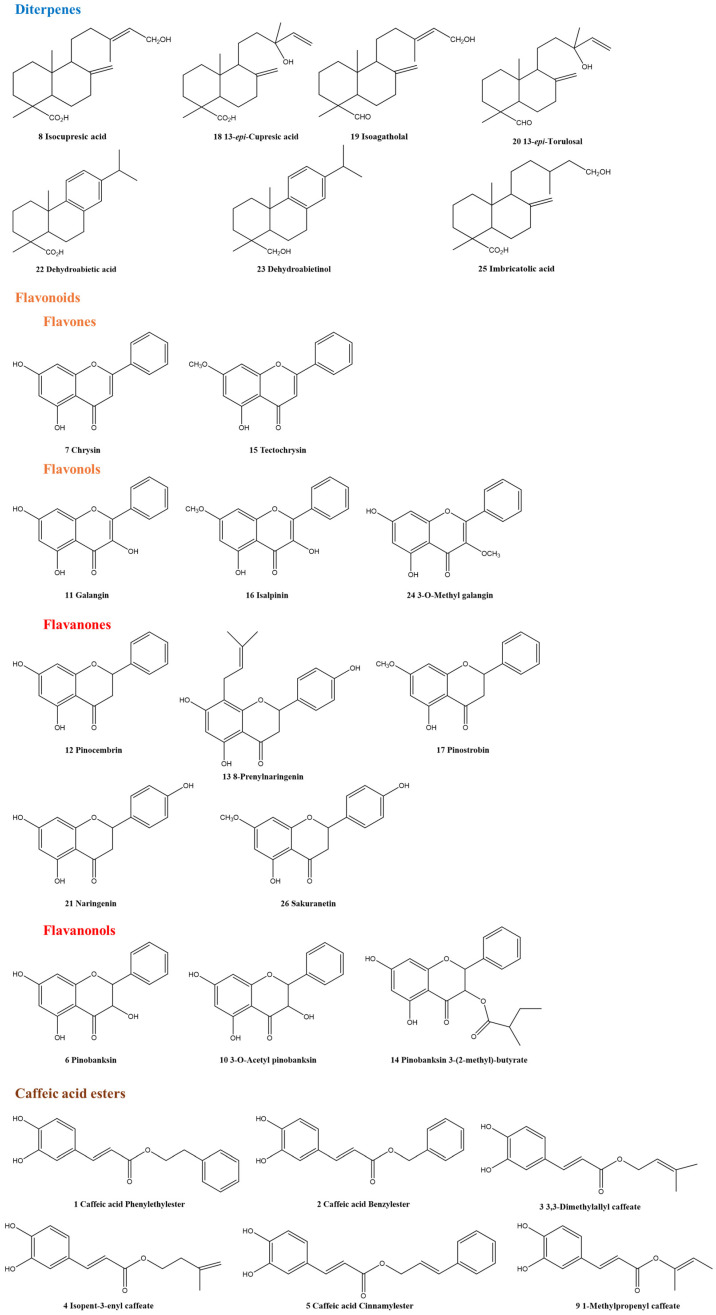
Chemical structures of the 26 secondary metabolites identified in PR09 propolis methanolic extract by NMR-HetCA analysis, comprising 7 diterpenes, 13 flavonoids and 6 caffeic acid esters. Compound identification was achieved prior to isolation through interpretation of NMR-HetCA total and partial correlation plots, supported by STOCSY analysis. Compound numbering corresponds to that used throughout the text and in Table S3, which lists all identified compounds alongside their classification as active (red) or inactive (blue) contributors to DPPH and collagenase inhibitory activity, based on total and partial NMR-HetCA correlation plots.

NMR-HetCA was applied to correlate the antioxidant and collagenase inhibitory activity of the fractions with their spectroscopic fingerprint. The 43 FCPC fractions were forwarded for ^1^H-NMR analysis and then processed in a MATLAB environment (bucketing of the spectra and correlation with their anti-collagenase and antioxidant activity, respectively). Analysis of the total and partial NMR-HetCA plots revealed 26 secondary metabolites, including 7 diterpenes, 13 flavonoids and 6 caffeic acid esters, as shown in Figure 5 and Table S3. NMR structure elucidation of compounds was based on comparison with literature data.

### 2.6. NMR-HetCA and Collagenase Activity

**Figure 6:**
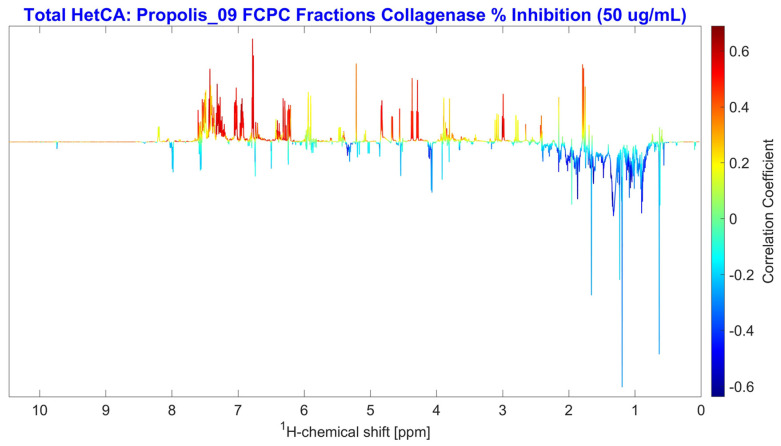
Total NMR-HetCA correlation plot of the ^1^H NMR spectral profiles of the 43 final FCPC fractions against their collagenase inhibitory activity (% inhibition at 50 μg/mL). Positive correlations (red peaks) indicate spectral signals associated with metabolites contributing to collagenase inhibition, while negative correlations (blue peaks) correspond to metabolites that do not contribute to the observed activity.

The correlation between anti-collagenase activity and the NMR spectra of all fractions was examined using NMR-HetCA. The resulting total coefficient plot (total HetCA plot) (Figure 6) indicated that the activity correlated with phenolic compounds, as reflected by positive correlations (red peaks at the aromatic region of spectrum), whereas terpenoids (blue-aliphatic region) seemed not to contribute at all.

**Figure 7:**
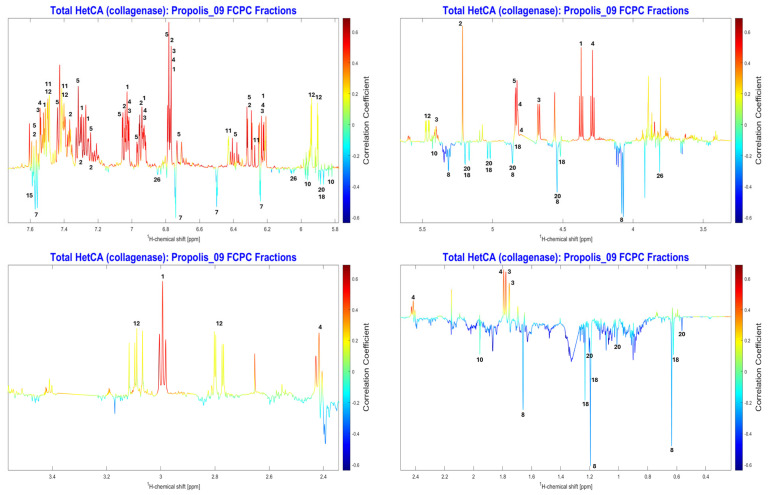
Expanded regions of the total NMR-HetCA correlation plot (Figure 6) highlighting the ^1^H NMR signals used for the identification of secondary metabolites contributing (red peaks, positive correlation) or not contributing (blue peaks, negative correlation) to the collagenase inhibitory activity of PR09 propolis fractions. Compound numbers correspond to the structures shown in Figure 5 and the assignments listed in Table S3, enabling unambiguous assignment of key diagnostic signals to individual metabolites prior to isolation.

Detailed analysis of the total NMR-HetCA plot (Figure 7), showed that caffeic acid derivatives (compounds **1**–**5**) were highly correlated and predicted to be the most active collagenase inhibitors. Furthermore, galangin (**11**) and pinocembrin (**12**) also contributed significantly to the anti-collagenase activity of the extract. In contrast, terpenoids like isocupressic acid (**8**) and 13-*epi*-cupressic acid (**18**) seemed to have no correlation with the collagenase inhibition. The peaks of chrysin (**7**), a flavonoid, were also observed in the negative region (blue peaks) of the total NMR-HetCA plot, indicating a lack of contribution to the observed activity.

To further assist compound identification, the correlations observed in consecutive sets of four fractions (quadruplets) were also examined, and a series of pseudospectra—presented as partial NMR-HetCA plots—were generated accordingly. A total of 40 partial NMR-HetCA plots were obtained for the 43 fractions of PR09 methanolic extract in correlation with their collagenase inhibitory activities, revealing potential bioactive metabolites. For instance, the partial NMR-HetCA plot of fractions Pr09_Fr21-29 (Figure S3) revealed the presence of pinocembrin (**12**) and 13-*epi*-cupresic acid (**18**), whereas peaks in the NMR-HetCA plot for the fractions Pr09_Fr25-30 (Figure S4) corresponded to galangin (**11**) and 3-O-methyl galangin (**24**). In addition, STOCSY analysis [17] facilitated the identification of several metabolites, such as pinocembrin (**12**) and 13-*epi*-cupresic acid (**18**) (Figure S5).

### 2.7. NMR-HetCA and DPPH Activity

**Figure 8:**
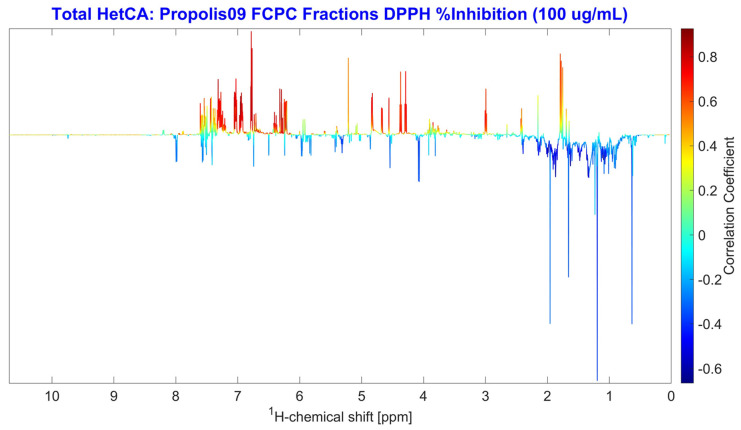
Total NMR-HetCA correlation plot of the ^1^H NMR spectral profiles of the 43 final FCPC fractions against their DPPH free radical scavenging activity (% inhibition at 100 μg/mL). Positive correlations (red peaks) indicate spectral signals associated with metabolites contributing to DPPH radical scavenging, while negative correlations (blue peaks) correspond to metabolites that do not contribute to the observed activity.

The correlation between the antioxidant activity and the NMR spectra of all fractions was examined using NMR-HetCA. The resulting total coefficient plot (Figure 8) indicated that antioxidant activity also correlated with phenolic compounds, while terpenoids showed no apparent contribution, as expected.

**Figure 9:**
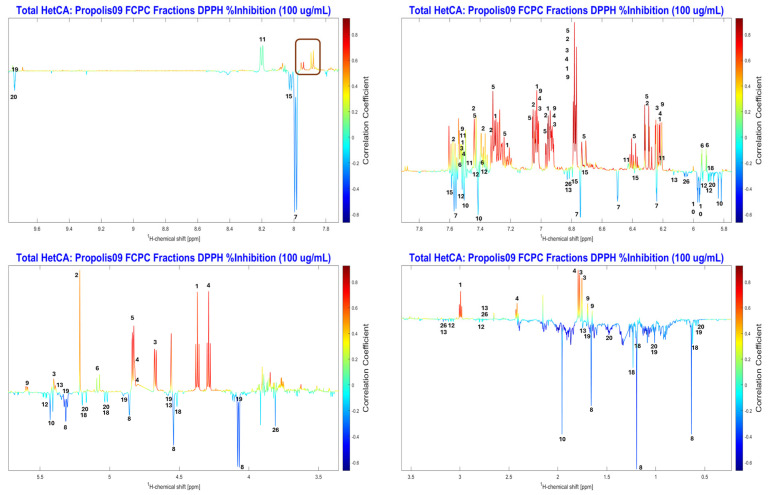
Expanded regions of the total NMR-HetCA correlation plot (Figure 8) highlighting the ^1^H NMR signals used for the identification of secondary metabolites contributing (red peaks, positive correlation) or not contributing (blue peaks, negative correlation) to the DPPH free radical scavenging activity of PR09 propolis fractions. Compound numbers correspond to the structures shown in Figure 5 and the assignments listed in Table S3.

The total heterocovariance plot predicted caffeic acid derivatives (compounds **1**, **3**–**5**, **9**) to be the most potent DPPH radical scavengers. Galangin (**11**), caffeic acid benzyl ester (**2**) and pinobanksin (**6**) also appeared to contribute, but with more moderate activity. In contrast, isocupressic acid (**8**), 13-*epi*-cupressic acid (**18**), 13-*epi*-torulosal (**20**), pinocembrin (**12**), 3-O-acetyl-pinobanskin (**10**) and chrysin (**7**) were observed in the negative region of the total NMR-HetCA plot (Figure 9), indicating that they had no correlation with antioxidant activity.

Accordingly, compound identification was further supported by examining partial NMR-HetCA plots in parallel with STOCSY analysis. For instance, the partial NMR-HetCA plot of fractions Pr09_Fr53-56 (Figure S6) revealed the presence of caffeic acid phenethyl ester (**1**), 3,3-dimethylallyl caffeate (**3**), isopent-3-enyl caffeate (**4**), caffeic acid cinnamyl ester (**5**) and 1-methylpropenyl caffeate (**9**). In addition, STOCSY [14] facilitated the identification of several metabolites, such as galangin (**11**) and pinobanksin (**6**) (Figure S7).

Caffeic acid phenethyl ester (CAPE, commercially obtained), along with five additional compounds previously isolated in our laboratory, were evaluated for their bioactivities (Table 1) in order to validate the predictive accuracy of the NMR-HetCA analysis.

Compound **1** (caffeic acid phenethyl ester-CAPE) showed strong DPPH inhibition at both concentrations with percentages of 92.54 at 200 μg/mL and 88.35 at 100 μg/mL, whereas galangin (**11**) demonstrated moderate activity 51.44% DPPH inhibition at 200 μg/mL while isocupressic acid (**8**), 13-*epi*-cupressic acid (**18**) and chrysin (**7**) showed very low DPPH scavenging capacity. These results were in accordance with NMR-HetCA indications.

Regarding collagenase inhibition, the secondary metabolites galangin (**11**) and pinocembrin (**12**) showed significant inhibition with percentages of 66.91% and 73.34%, respectively, at 100 μg/mL, while 13-*epi*-cupressic acid (**18**) showed very low activity. These results further validated the effectiveness of the NMR-HetCA predictive capability. However, chrysin (**7**) and CAPE (**1**) exhibited moderate inhibition of collagenase (51.85% and 50.11% at 100 μg/mL, respectively), results that have a deviation from the activity predicted by studying the total NMR-HetCA plot. In particular, as shown in Figure 7, chrysin (**7**) appeared as non-contributing to the activity (negatively correlated, light blue colored) whereas CAPE (**1**) was predicted as highly contributing (positively correlated, red colored). In order to explain this discrepancy, the partial NMR-HetCA plots were further investigated. By observing partial NMR-HetCA plot of fractions PR09_Fr46-49 and PR09_Fr49-52 (Figure 9 and Figures S8 and S9, respectively), the peaks of chrysin (**7**) could be tracked, appearing as contributing and non-contributing, respectively. From Figure S9, it is also observed that in fractions PR09_Fr49-52, caffeic acid derivatives (**1–5**, **9**) were co-eluting. This co-elution of chrysin with the active caffeic acid derivatives could be the reason of these misleading results. In the case where caffeic acid derivatives were not present (NMR-HetCA plot PR09_Fr46-49), the peaks of chrysin showed an important correlation with anti-collagenase activity.

As previously demonstrated by our group in the in-depth evaluation of NMR-HetCA using standard mixtures [13], this approach can occasionally yield misleading results (false negative and false positive identification of bioactives) due to the co-elution of active compounds at low concentrations with more active compounds at a higher concentration or because of phenomena of cumulative and/or synergistic activity of the co-eluted substances.

**Figure 10:**
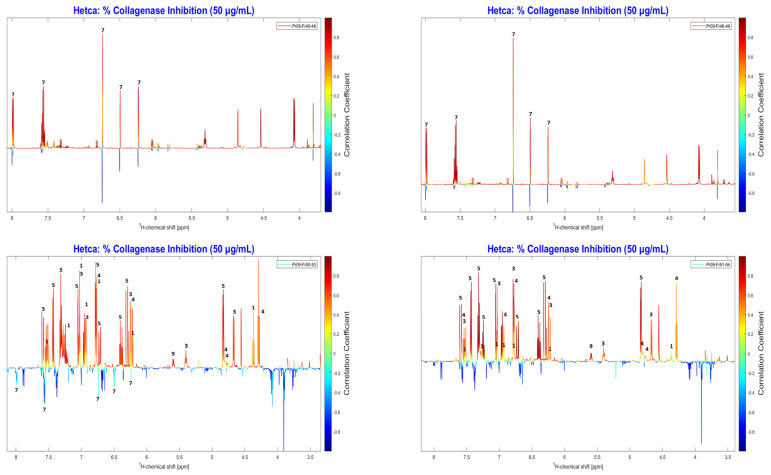
Expanded regions of partial NMR-HetCA correlation plots derived from selected fraction subsets (Pr09_Fr45–58, Pr09_Fr46–49, Pr09_Fr50–53 and Pr09_Fr51–54) of PR09 propolis, highlighting ^1^H NMR signals used for the identification of secondary metabolites contributing (red peaks, positive correlation) or not contributing (blue peaks, negative correlation) to the collagenase inhibitory activity. Partial NMR-HetCA analysis of fraction subsets enables higher resolution discrimination of co-eluting metabolites whose contributions may be masked in the total correlation plot (Figure 6) by exploiting localized concentration gradients within the fractionation sequence. Compound numbers correspond to the structures shown in Figure 5.

Accordingly, the deviation on the prediction of CAPE (**1**) activity is attributed to its co-elution with more active caffeic acid derivatives. Taking a much closer look in Figure 10, from the partial NMR-HetCA plots of fractions PR09_Fr50-53 and PR09_Fr51-54, the peaks of CAPE (**1**) seemed to have a lower contribution to the anti-collagenase activity (yellow coloring) compared to other caffeic acid derivatives’ peaks (highly correlated, red coloring).

## 3. Discussion

NMR-HetCA can serve as a method of choice for the rapid detection and identification of the majority of active components in a complex mixture prior to their isolation. In the case of PR09, NMR-HetCA offered significant advantages regarding experimental duration over traditional isolation methods. While conventional bioactivity-guided isolation may require a long time to be completed, NMR-HetCA enabled reliable bioactivity predictions within only a few days of obtaining the FCPC fractions and NMR data. This rapid and efficient identification capability facilitates the prioritization of isolation efforts on the most promising bioactive compounds from other propolis samples rather than pursuing exhaustive isolation procedures. Effective detection of bioactive secondary metabolites through NMR-HetCA requires careful fractionation of the extract, ensuring sufficient variation in compound concentrations across fractions, since bioactivity is often concentration-dependent. FCPC technique was selected for the fractionation due to its lack of solid stationary phase, broader elution range, high percentage of recovery, wide range of solvent choice and high separation capability when the proper solvent system is selected [9,18].

The value of integrating NMR spectroscopy with chemometric tools for the investigation of complex natural matrices has been extensively recognized in the literature. As highlighted by Rebiai et al. [19], NMR-based metabolomics can be utilized to assess the composition of plant material quickly and reliably, with the combination of NMR and chemometrics offering a powerful strategy for both qualitative profiling and quantitative assessment of bioactive constituents. NMR is a fast and accurate analytical method, and becomes increasingly important when associated with chemometrics, particularly for the resolution of complex analytical problems in natural product and pharmaceutical research [20]. The present findings align well with this broader consensus: the application of NMR-HetCA to the FCPC fractions of PR09 allowed for the simultaneous correlation of spectroscopic fingerprints with two independent bioactivity assays—anti-collagenase and DPPH radical scavenging activity—demonstrating precisely the type of multivariate approach advocated in literature.

The NMR-HetCA methodology also addresses one of the persistent challenges in bioactivity-guided natural product research, namely the time and consumables cost of exhaustive fractionation–isolation cycles. Traditional natural product identification relies on extensive fractionation and time-consuming structural elucidation steps. By contrast, the approach applied here circumvents much of this burden by correlating the spectroscopic profiles of fractions directly with their biological activity, enabling the early prioritization of promising metabolites and giving the possibility to focus only on the targeted isolation of novel compounds. This is consistent with the concept of bioactivity-driven NMR prioritization, which has been increasingly applied in drug discovery contexts to narrow down candidate compounds before committing to intensive isolation [21]. In this study, the bioactive compounds identified in PR09 through NMR-HetCA are consistent with those previously reported for poplar-type European propolis using conventional chromatographic and bioactivity-guided methods, including caffeic acid derivatives, CAPE, galangin and pinocembrin, lending strong validation to the approach. In particular, CAPE has been extensively documented as the principal bioactive constituent of propolis, while galangin and pinocembrin have been isolated and confirmed as active flavonoids from European propolis samples of diverse geographic origins, through laborious chromatographic isolation requiring weeks to months of work [22,23,24,25,26]. The collagenase inhibitory activity observed here for caffeic acid derivatives, chrysin, pinocembrin and galangin is consistent with their broader MMP-modulating properties reported in the literature (especially MMP-2, MMP-3, MMP-8, MMP-9 and MMP-13) and supports their plausible contribution to collagen-degrading MMP inhibition in poplar-type propolis extracts [27,28,29,30,31]. For pinocembrin, direct MMP inhibition has not been clearly demonstrated in the literature. The fact that NMR-HetCA rapidly reproduced, from a single Greek sample, the same bioactivity compound associations established through decades of conventional isolation studies across multiple geographic origins, underscores its value as a high-throughput dereplication and prioritization tool—particularly for less-studied natural products where no literature yet exists.

Also, as noted in from our previous work, the predictive power of correlation-based NMR approaches is inherently subject to limitations arising from co-elution phenomena [9]. Moreover, the complex natural product matrices, such as plant extracts, appear in NMR spectra with considerable signal overlap, which can complicate the extraction of unambiguous information, and likewise, the co-elution of bioactive compounds at varying concentrations can produce both false negative and false positive activity predictions, as was observed here for chrysin (**7**) and CAPE (**1**). The role of chemometrics in quality control and authenticity of pharmaceutical and natural products will definitely increase in the future, and the refinement of correlation-based platforms such as NMR-HetCA—particularly through the integration of partial HetCA plots and STOCSY analysis—represents a step toward more robust and interpretable bioactivity prediction from complex mixtures. The complementary use of these tools, as demonstrated in the present work, offers a realistic framework for deconvoluting co-elution artefacts and improving the reliability of bioactive metabolite identification prior to isolation.

## 4. Materials and Methods

### 4.1. Solvents and Reagents

Analytical grade methanol and n-heptane for propolis extraction, as well as dichloromethane, ethyl acetate, sulfuric acid (H_2_SO_4_) and vanillin for HPTLC analysis and ethanol for bioassays were purchased from Merck (Merck, Darmstadt, Germany). 2,2-Diphenyl 1-picrylhydrazyl (DPPH) and potassium persulfate (K_2_S_2_O_8_) were purchased from Sigma-Aldrich (Sigma Aldrich, Steinheim, Germany). For collagenase inhibition assay, collagenase from *Clostridium histolyticum* (0.25–1.0 FALGPA units/mg solid), Tris(hydroxymethyl)aminomethane hydrochloride (Trizma−HCl) and phosphoramidon disodium salt were bought from Sigma-Aldrich. Caffeic acid phenethyl ester (CAPE) was purchased from Cayman Chemical Company (Ann Arbor, MI, USA).

### 4.2. Sample Collection and Extract Preparation

Propolis sample 9 (code PR09) was collected from Mt. Olympus, Macedonia. Prior to the extraction, the sample was grounded and then 4 g were initially defatted with n-heptane (2 × 20 mL). Subsequently, the remaining material (~2.8 g) was extracted in an ultrasonic bath with methanol (2 × 20 mL). The two resulting methanolic extracts were merged and concentrated to dryness by a rotary evaporator (Büchi, Essen, Germany), under vacuum. The dry extract was stored at −20 °C until analysis.

### 4.3. Fast Centrifugal Partition Chromatography (FCPC)

The fractionation of PR09 methanolic extract was performed by FCPC (KROMATON, Annonay, France) with a 200 mL column, operating at 500 rpm in descending mode for filling the column and 1500 rpm in ascending mode for the elution. The system was equipped with a LabAlliance pump (LabAlliance, State College, PA, USA) at a flow rate of 25 mL/min for filling the column and 10 mL/min for the elution and a BUCHI C-660 fraction collector (Büchi Labortechnik AG, Flawil, Switzerland) set to collect 12 mL per fraction. System Q (heptane/ethyl acetate/methanol/water: 3/2/3/2) from the ARIZONA family systems [10] was selected for the fractionation. A total of 600 mg of dry extract, dissolved in 10 mL of the stationary phase, was injected. In total, 59 fractions were collected and then submerged into 43 resulting fractions, which were evaporated under reduced pressure using a rotary evaporator (Büchi, Essen, Germany) and were forwarded for HPTLC profiling, 1H-NMR analysis and in vitro evaluation of their antioxidant capacity and collagenase inhibitory activity.

### 4.4. Isolation of Chemical Constituents

As part of our ongoing systematic studies on propolis from various Greek geographic regions [32], several metabolites previously isolated from different propolis samples have been deposited in our laboratory and are routinely used as internal standards. For the present study, isocupressic acid (**8**), 13-*epi*-cupressic acid (**18**), pinocembrin (**12**), galangin (**11**) and chrysin (**7**) were employed in the biological assays.

### 4.5. Evaluation of Free Radical Scavenging Activity by DPPH Assay

The DPPH assay was used for biological screening for the methanolic extract, with its FCPC fractions and pure metabolites as previously described by Lee et al. [33] with minor modifications. The absorbance was measured at 517 nm, using a Tecan Infinite M1000 PRO microplate reader (Tecan GmbH, Grödig, Austria), while the system was operating under the Tecan i-control v. 1.11. All evaluations were performed in triplicate, while gallic acid was used as the positive control (IC_50_ = 30.2 μM). The in vitro DPPH inhibition assay for the extract and the pure compounds was performed at 200 μg/m and 100 μg/mL (final concentration), whereas for the fractions, it was performed at the final concentration of 100 μg/mL.

### 4.6. Collagenase Inhibitory Activity

Collagenase inhibitory activity was examined using a slightly modified method described by Teramachi et al. [34]. Briefly, the test samples, enzyme solution and Tris-HCl buffer (pH 7.3) were added to 96-well microtiter plate and preincubated for 10 min at 37 °C. Afterwards, the substrate solution was added to initiate the reaction. The fluorescence values were measured at an excitation of 320 nm and an emission of 405 nm after 30 min incubation at 37 °C. These assays were performed in triplicate using phosphoramidon as a positive control. The inhibition ratio of the samples was calculated by comparing the fluorescence increase produced by the sample with that of the negative control.

### 4.7. HPTLC Chemical Profiling

For the chemical profiling of the FCPC fractions, the samples were applied on HPTLC plate silica gel 60, 20 × 10 cm (Merck, Germany), as 8 mm bands, using an automatic TLC Sampler 4 (ATSA-4, CAMAG, Muttenz, Switzerland). The chromatographic separation was performed in the Automatic Developing Chamber 2 (ADC 2) with a mixture of dichloromethane and methanol (90:10 *v*/*v*), up to a migration distance of 80 mm (from the lower plate edge). The plate was pre-saturated for 5 min with the mobile phase before plate development and dried automatically for 5 min after plate development. The plates were scanned (254 and 366 nm) with the CAMAG TLC Scanner 4 (CAMAG, Muttenz, Switzerland) and documented under UV 254 and 366 nm and after spraying with sulfuric vanillin using the TLC Visualizer 2 (CAMAG, Muttenz, Switzerland). The HPTLC plate images were exported from the winCATS software v2.1.

### 4.8. NMR-HeteroCovariance Approach (NMR-HetCA)

NMR experiments were conducted on a 600 MHz Avance III Brucker NMR Spectrometer (Bruker, Ettlingen, Germany) equipped with a 5 mm double resonance broadband inverse (BBI) detection probe. Fractions derived from FCPC were diluted in methanol-*d*_4_ with 0.03% TMS purchased from Eurisotop (Euriso-Top GmbH, Saarbrücken, Germany) and transferred to 5 mm NMR tubes (LabScape, Bruker, Ettlingen, Germany). The NMR spectra were acquired at 298 K, after a 5 min resting period for temperature stabilization. Data acquisition and processing were done with Bruker TopSpin 3.7. Metabolic profiling 1D NMR spectra (noesygppr1d) were acquired using water suppression with d1 = 6 s; AQ = 2.7 s; FID data points = 64 k; SW = 20 ppm; ns = 256. The transmitter offset was set manually to achieve optimal suppression of the residual water signal. FIDs were zero-filled and multiplied by an exponential weighting function corresponding to a line broadening of 0.3 Hz before Fourier transformation. Then MATLAB 2024a environment was used for the bucketing of the spectra and the correlation to their activities, through HetCA toolbox, as previously described [9]. In addition to the correlation of all fractions, the procedure was also performed using repeats of four subsequent spectra each time, which is the minimum number of spectra required to achieve a successful correlation. The resulting bucket-specific covariance values were presented as an NMR pseudospectrum (HetCA plot), where each point was color-coded according to the respective correlation values.

## Figures and Tables

**Table 1:** DPPH and collagenase % inhibition activity of standard compounds in two different concentrations.

Metabolites	DPPH % Inhibition (C = 200 μg/mL) ± SD	DPPH % Inhibition (C = 100 μg/mL) ± SD	Collagenase % Inhibition (C = 100 μg/mL) ± SD	Collagenase % Inhibition (C = 50 μg/mL) ± SD
Isocupressic acid	8.49 ± 0.55	not tested	not tested	2.15 ± 1.88
13-*epi*-cupressic acid	6.58 ± 2.37	not tested	not tested	19.13 ± 2.33
pinocembrin	12.57 ± 0.31	not tested	73.34 ± 1.35	47.56 ± 0.78
galangin	51.44 ± 0.54	35.23 ± 0.99	66.91 ± 2.99	47.55 ± 2.69
chrysin	13.95 ± 0.76	3.03 ± 0.37	51.85 ± 1.65	43.75 ± 0.56
CAPE	92.54 ± 0.16	88.35 ± 0.14	50.11 ± 0.18	30.48 ± 0.98

## Data Availability

All data are available upon request from the authors.

## Supplementary Materials

The following supporting information can be downloaded at: https://www.mdpi.com/article/10.3390/molecules31101742/s1, Figure S1: HPTLC profiling of all 59 FCPC fractions of PR09 propolis methanolic extract on normal-phase silica gel plates, developed with dichloromethane:methanol (90:10). Documentation was performed under UV light at 254 nm (top), revealing flavonoids and phenolic acid derivatives by absorbance, and under white light after derivatization with vanillin-sulfuric acid reagent followed by heating (bottom), enabling detection of terpenes as blue-purple spots and flavonoids as yellow-orange spots. Based on the observed chemical profiles and concentration gradients across fractions, the initial 59 fractions were merged into 43 final fractions (indicated by bracketing), balancing chemical diversity with adequate concentration variation required for NMR-HetCA analysis; Figure S2: Stack plot of NMR spectra of FCPC fractions in methanol-*d_4_*; Figure S3: Zoom in regions of partial NMR-HetCA plot (Pr09_Fr21-29) and identification of secondary metabolites (corresponding numbers) contributing (red peaks) or not (blue) in the collagenase inhibitory activity; Figure S4: Zoom in regions of partial NMR-HetCA plot (PR09_Fr25-30) and identification of secondary metabolites (corresponding numbers) contributing (red peaks) or not (blue) in the collagenase inhibitory activity; Figure S5: Identification of pinocembrin (**12**) (left) and 13-epi-cupresic acid (**18**) (right) through STOCSY based on selected signals (5.4785 and 4.5170 ppm, respectively); Figure S6: Zoom in region of partial NMR-HetCA plot (Pr09_Fr53-56) and identification of secondary metabolites (corresponding numbers) contributing (red peaks) or not (blue) in the collagenase inhibitory activity; Figure S7: Identification of galangin (**11**) (left) and pinobaskin (**6**) (right) through STOCSY based on selected signals (8.1935 and 5.0735 ppm, respectively); Figure S8: Zoom in region of partial NMR-HetCA plot (Pr09_Fr46-49) and identification of secondary metabolites (corresponding numbers) contributing (red peaks) or not (blue) in the collagenase inhibitory activity; Figure S9: Zoom in region of partial NMR-HetCA plot (Pr09_Fr49-52) and identification of secondary metabolites (corresponding numbers) contributing (red peaks) or not (blue) in the collagenase inhibitory activity; Table S1: Collagenase % inhibition of all FCPC fractions from in vitro assay at concentration 50 μg/ml; Table S2: DPPH % inhibition of all FCPC fractions from in vitro assay at concentration 100 μg/ml; Table S3: List of compounds identified through total and partial NMR-HetCA plots regarding DPPH and collagenase activity. Compounds are colored red or blue according to their contribution or not to the activity, respectively.
