# Supplementary material for: Uncovering Bioactive Compounds in Propolis Extracts Prior to Isolation Through NMR Chemometric Analysis
Source: Molecules. 2026 May 20;31(10):1742. doi: 10.3390/molecules31101742 (PMC13209362; doi:10.3390/molecules31101742)
Supplement: Supplementary file 1 [file molecules-31-01742-s001.zip › molecules-4308607-supplementary.pdf]

molecules

Supplementary Materials

Uncovering Bioactive Compounds in Propolis Extracts Prior to Isolation Through NMR Chemometric Analysis

Maria-Ioanna Stavropoulou, Antigoni Cheilari *, Konstantia Graikou, Ioanna Chinou and Nektarios Aligiannis

Laboratory of Pharmacognosy and Natural Products Chemistry, Faculty of Pharmacy, National and
Kapodistrian University of Athens, Panepistimiopolis Zografou, 15771 Athens, Greece;
mstavropoul@yahoo.gr (M.I.S.); kgraikou@pharm.uoa.gr (K.G.); ichinou@pharm.uoa.gr (I.C.);
aligiannis@pharm.uoa.gr (N.A.)
* Correspondence: cheilarianti@pharm.uoa.gr

Figures

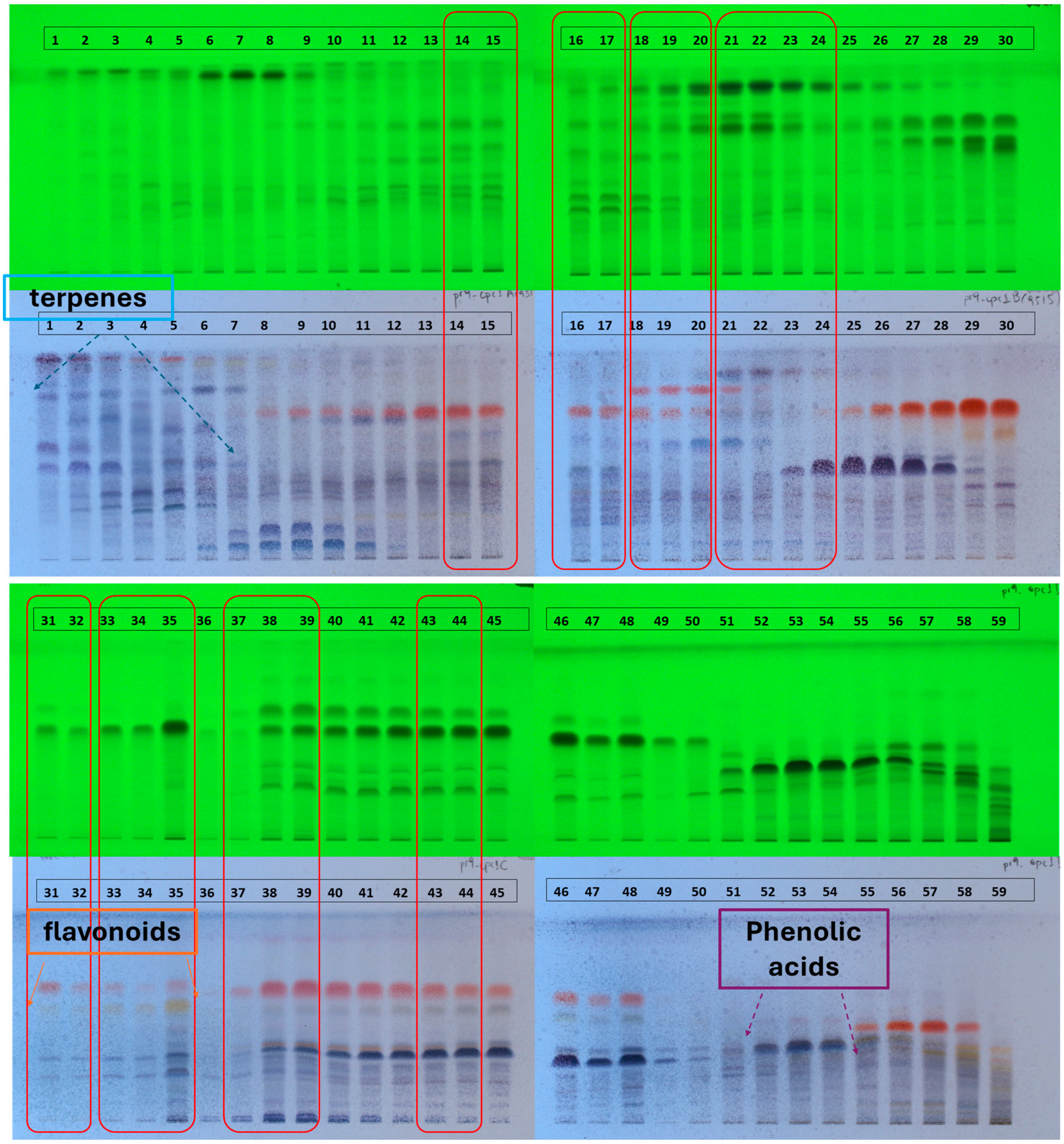

Figure S1. HPTLC profiling of all 59 FCPC fractions of PR09 propolis methanolic extract on normal-phase silica gel plates, developed with dichloromethane:methanol (90:10). Documentation was performed under UV light at 254 nm (top), revealing flavonoids and phenolic acid derivatives by absorbance, and under white light after derivatization with vanillin-sulfuric acid reagent followed by heating (bottom), enabling detection of terpenes as blue-purple spots and flavonoids as yellow-orange spots. Based on the observed chemical profiles and concentration gradients across fractions, the initial 59 fractions were merged into 43 final fractions (indicated by bracketing), balancing chemical diversity with adequate concentration variation required for NMR-HetCA analysis.

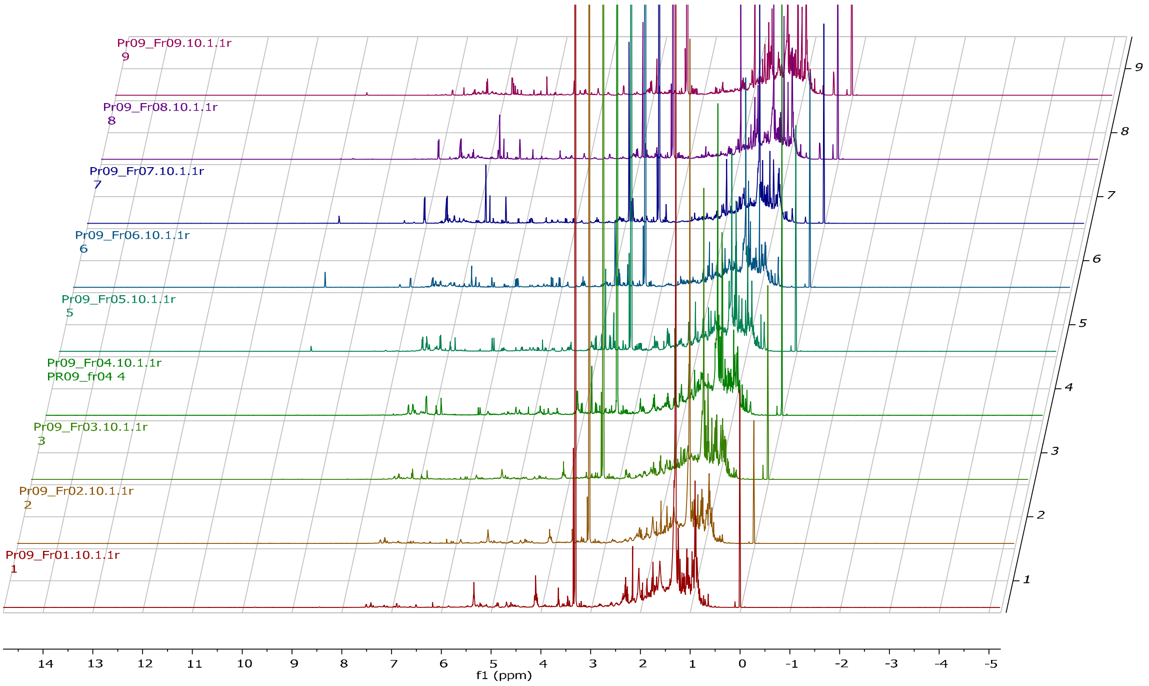
Pr09_Fr09.10.1.1r
9
Pr09_Fr08.10.1.1r
8
Pr09_Fr07.10.1.1r
7
Pr09_Fr06.10.1.1r
6
Pr09_Fr05.10.1.1r
5
Pr09_Fr04.10.1.1r
PR09_fr04 4
Pr09_Fr03.10.1.1r
3
Pr09_Fr02.10.1.1r
2
Pr09_Fr01.10.1.1r
1
14 13 12 11 10 9 8 7 6 5 4 3 2 1 0 -1 -2 -3 -4 -5
f1 (ppm)

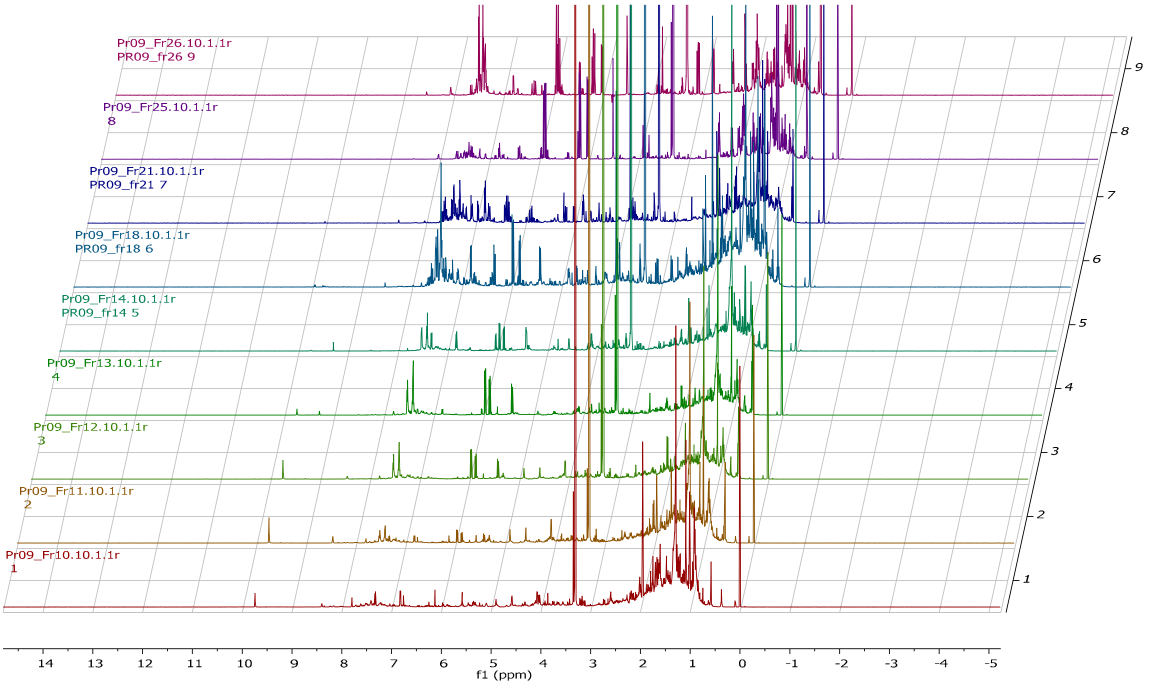
Pr09_Fr26.10.1.1r
PR09_fr26 9
Pr09_Fr25.10.1.1r
8
Pr09_Fr21.10.1.1r
PR09_fr21 7
Pr09_Fr18.10.1.1r
PR09_fr18 6
Pr09_Fr14.10.1.1r
PR09_fr14 5
Pr09_Fr13.10.1.1r
4
Pr09_Fr12.10.1.1r
3
Pr09_Fr11.10.1.1r
2
Pr09_Fr10.10.1.1r
1
14 13 12 11 10 9 8 7 6 5 4 3 2 1 0 -1 -2 -3 -4 -5
f1 (ppm)

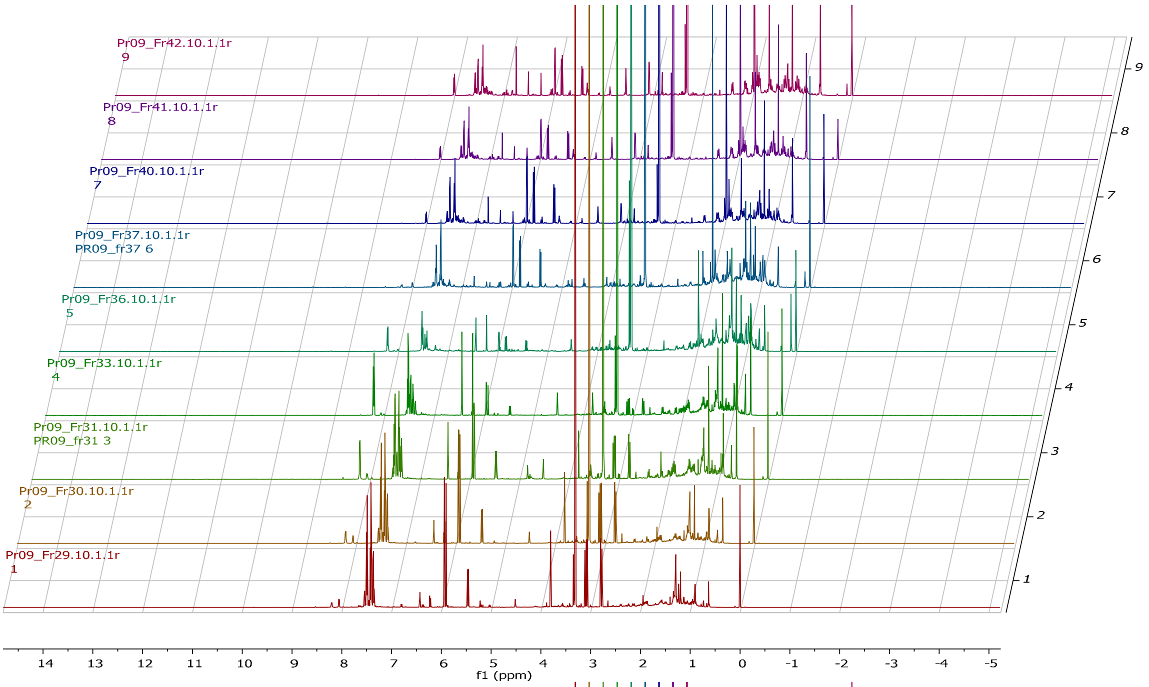
Pr09_Fr42.10.1.1r
9
Pr09_Fr41.10.1.1r
8
Pr09_Fr40.10.1.1r
7
Pr09_Fr37.10.1.1r
PR09_fr37 6
Pr09_Fr36.10.1.1r
5
Pr09_Fr33.10.1.1r
4
Pr09_Fr31.10.1.1r
PR09_fr31 3
Pr09_Fr30.10.1.1r
2
Pr09_Fr29.10.1.1r
1
14 13 12 11 10 9 8 7 6 5 4 3 2 1 0 -1 -2 -3 -4 -5
f1 (ppm)

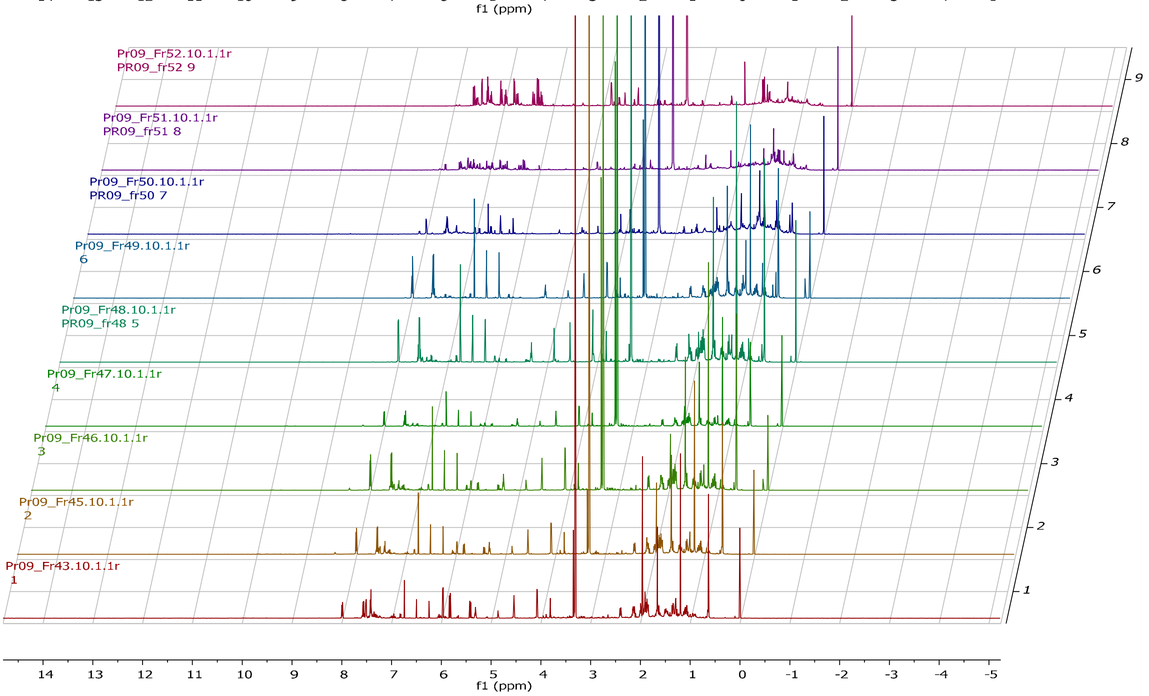
Pr09_Fr52.10.1.1r
PR09_fr52 9
Pr09_Fr51.10.1.1r
PR09_fr51 8
Pr09_Fr50.10.1.1r
PR09_fr50 7
Pr09_Fr49.10.1.1r
6
Pr09_Fr48.10.1.1r
PR09_fr48 5
Pr09_Fr47.10.1.1r
4
Pr09_Fr46.10.1.1r
3
Pr09_Fr45.10.1.1r
2
Pr09_Fr43.10.1.1r
1
14 13 12 11 10 9 8 7 6 5 4 3 2 1 0 -1 -2 -3 -4 -5
f1 (ppm)

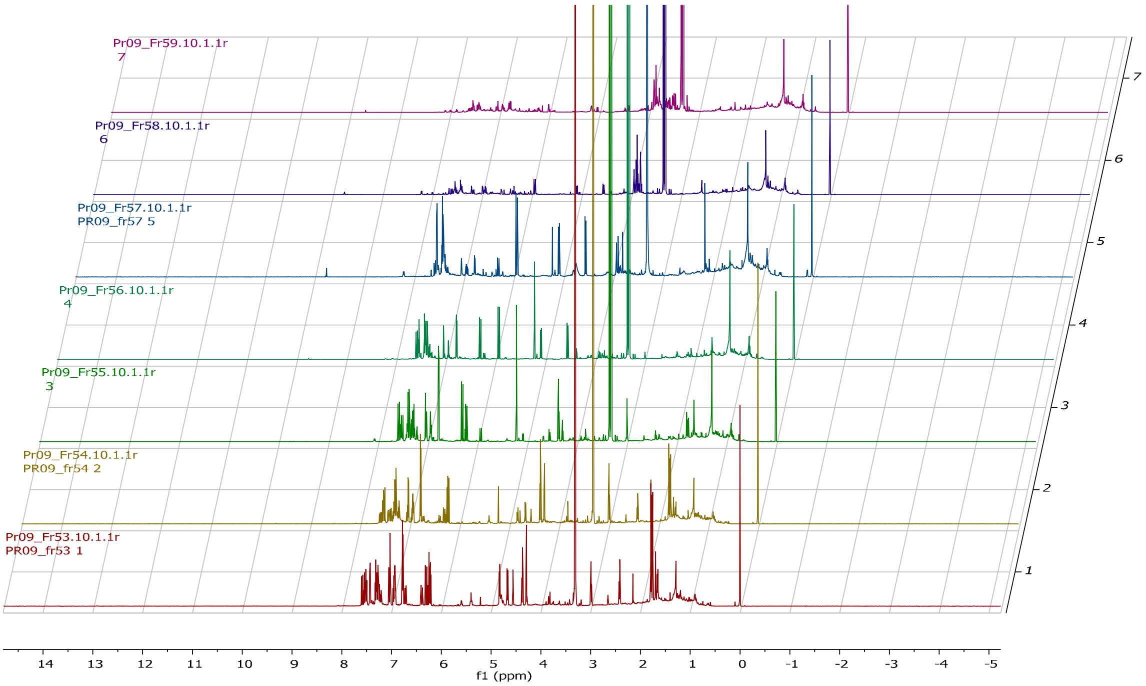

Figure S2. Stack plot of NMR spectra of FCPC fractions in methanol-d_4.

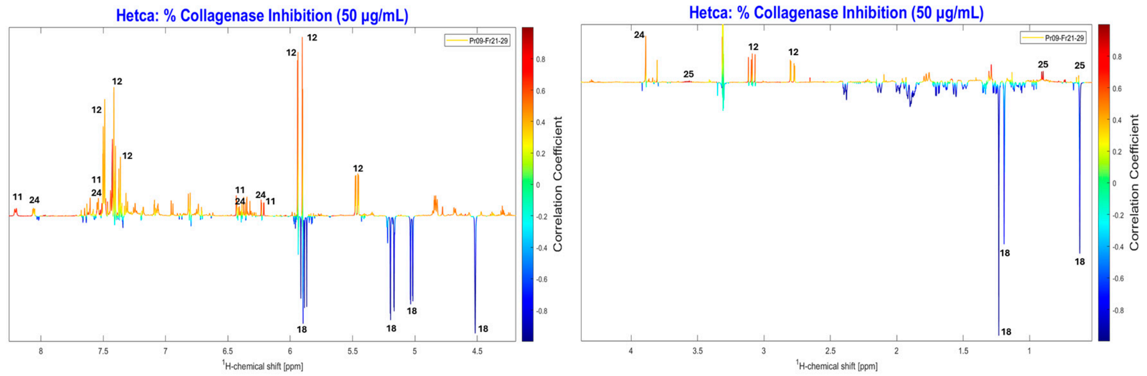

Figure S3. Zoom in regions of partial NMR-HetCA plot (Pr09_Fr21-29) and identification of secondary metabolites (corresponding numbers) contributing (red peaks) or not (blue) in the collagenase inhibitory activity.

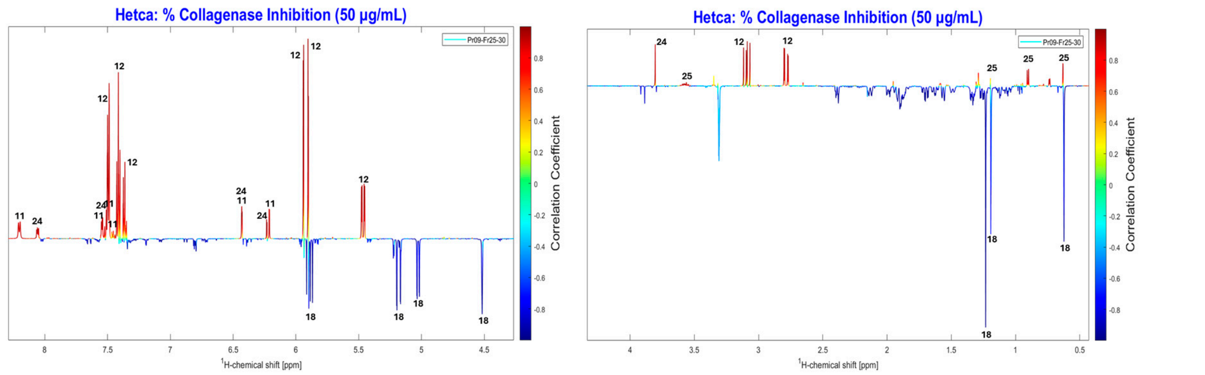

Figure S4. Zoom in regions of partial NMR-HetCA plot (PR09_Fr25-30) and identification of secondary metabolites (corresponding numbers) contributing (red peaks) or not (blue) in the collagenase inhibitory activity.

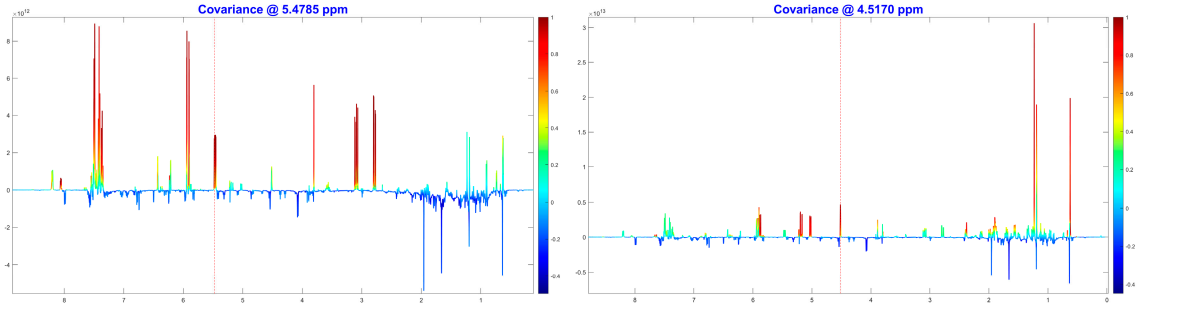

Figure S5. Identification of pinocembrin (12) (left) and 13-epi-cupresic acid (18) (right) through STOCSY based on selected signals (5.4785 and 4.5170 ppm, respectively).

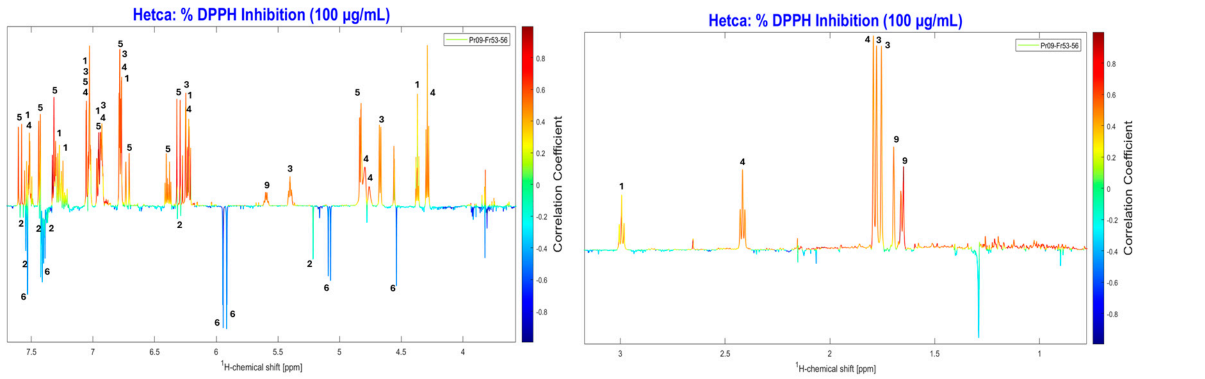

Figure S6. Zoom in region of partial NMR-HetCA plot (Pr09_Fr53-56) and identification of secondary metabolites (corresponding numbers) contributing (red peaks) or not (blue) in the collagenase inhibitory activity.

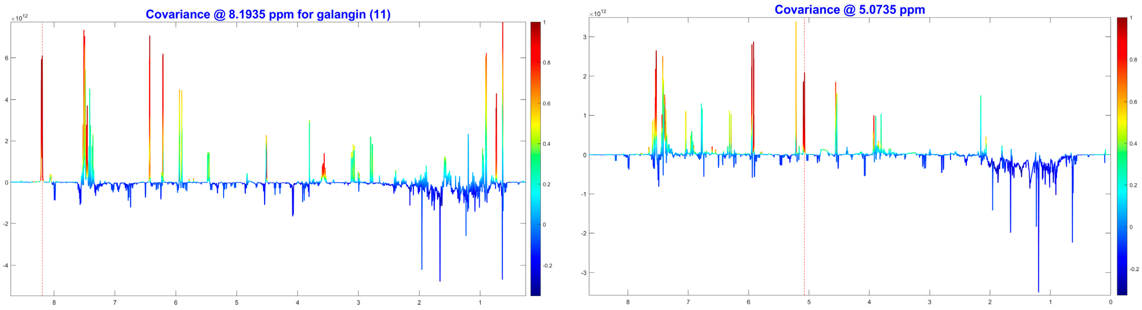

Figure S7. Identification of galangin (11) (left) and pinobaskin (6) (right) through STOCSY based on selected signals (8.1935 and 5.0735 ppm, respectively).

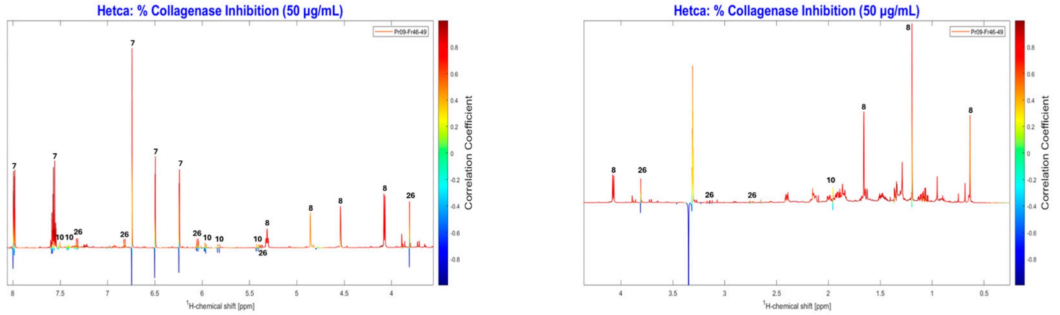

Figure S8. Zoom in region of partial NMR-HetCA plot (Pr09_Fr46-49) and identification of secondary metabolites (corresponding numbers) contributing (red peaks) or not (blue) in the collagenase inhibitory activity.

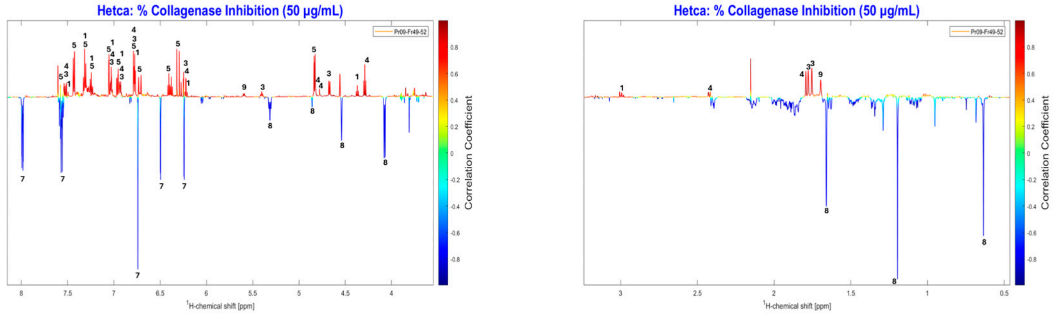

Figure S9. Zoom in region of partial NMR-HetCA plot (Pr09_Fr49-52) and identification of secondary metabolites (corresponding numbers) contributing (red peaks) or not (blue) in the collagenase inhibitory activity.

Tables

Table S1. Collagenase % inhibition of all FCPC fractions from in vitro assay at concentration 50 μg/ml.

A/A	Fraction Code	Collagenase inhibition % (50 μg/ml)
**1**	Pr09_Fr01	0.95±2.05
**2**	Pr09_Fr02	15.79±3.16
**3**	Pr09_Fr03	24.31±1.59
**4**	Pr09_Fr04	32.82±2.75
**5**	Pr09_Fr05	44.57±0.80
**6**	Pr09_Fr06	7.9±0.86
**7**	Pr09_Fr07	19.8±6.59
**8**	Pr09_Fr08	57.86±3.69
**9**	Pr09_Fr09	39.88±2.04
**10**	Pr09_Fr10	27.38±2.86
**11**	Pr09_Fr11	41.88±1.73
**12**	Pr09_Fr12	46.66±3.17
**13**	Pr09_Fr13	66.95±2.57
**14**	Pr09_Fr14	47.11±4.40
**15**	Pr09_Fr18	60.86±1.73
**16**	Pr09_Fr21	59.91±2.78
**17**	Pr09_Fr25	30.88±1.39
**18**	Pr09_Fr26	36.35±2.63
**19**	Pr09_Fr29	62.1±0.32
**20**	Pr09_Fr30	66.88±0.67
**21**	Pr09_Fr31	56.42±1.93
**22**	Pr09_Fr33	58.31±2.93
**23**	Pr09_Fr36	48.86±3.69
**24**	Pr09_Fr37	51.2±3.33
**25**	Pr09_Fr40	47.2±0.24
**26**	Pr09_Fr41	55.94±0.23
**27**	Pr09_Fr42	50.47±0.60
**28**	Pr09_Fr43	37.15±3.82
**29**	Pr09_Fr45	28.56±2.38
**30**	Pr09_Fr46	30.09±0.27
**31**	Pr09_Fr47	14.89±3.82
**32**	Pr09_Fr48	35.08±2.80
**33**	Pr09_Fr49	36.4±1.14
**34**	Pr09_Fr50	72.45±1.81
**35**	Pr09_Fr51	59.75±0.43
**36**	Pr09_Fr52	93.73±0.33
**37**	Pr09_Fr53	83.96±1.90
**38**	Pr09_Fr54	71.05±3.72
**39**	Pr09_Fr55	85.79±0.79
**40**	Pr09_Fr56	67.99±2.03

**41**	Pr09_Fr57	50.66±4.21
**42**	Pr09_Fr58	75.1±1.74
**43**	Pr09_Fr59	42.56±0.03

Table S2. DPPH % inhibition of all FCPC fractions from in vitro assay at concentration 100 μg/ml.

**A/A**	**Fraction Code**	**DPPH inhibition % (100 μg/ml)**
**1**	Pr09_Fr01	8.55±1.20
**2**	Pr09_Fr02	5.31±0.55
**3**	Pr09_Fr03	7.27±0.31
**4**	Pr09_Fr04	8.8±1.10
**5**	Pr09_Fr05	12.33±0.76
**6**	Pr09_Fr06	9.99±0.92
**7**	Pr09_Fr07	8.37±0.66
**8**	Pr09_Fr08	8.16±0.71
**9**	Pr09_Fr09	11.18±2.87
**10**	Pr09_Fr10	10.14±0.74
**11**	Pr09_Fr11	11.38±2.15
**12**	Pr09_Fr12	10.88±4.46
**13**	Pr09_Fr13	10.44±0.77
**14**	Pr09_Fr14	9.5±2.14
**15**	Pr09_Fr18	19.47±0.32
**16**	Pr09_Fr21	17.53±2.20
**17**	Pr09_Fr25	11.24±1.14
**18**	Pr09_Fr26	8.7±0.80
**19**	Pr09_Fr29	14.21±0.62
**20**	Pr09_Fr30	18.33±1.13
**21**	Pr09_Fr31	26.66±0.81
**22**	Pr09_Fr33	23.13±0.87
**23**	Pr09_Fr36	23.47±0.19
**24**	Pr09_Fr37	12.69±3.52
**25**	Pr09_Fr40	7.07±0.91
**26**	Pr09_Fr41	6.58±2.13
**27**	Pr09_Fr42	6.83±1.72
**28**	Pr09_Fr43	6.84±0.36
**29**	Pr09_Fr45	5.17±0.20
**30**	Pr09_Fr46	7.78±1.56
**31**	Pr09_Fr47	5.93±2.20
**32**	Pr09_Fr48	6.21±1.76
**33**	Pr09_Fr49	10.21±0.82

**34**	Pr09_Fr50	26.98±0.62
**35**	Pr09_Fr51	50.88±2.13
**36**	Pr09_Fr52	82.77±2.22
**37**	Pr09_Fr53	71.23±1.78
**38**	Pr09_Fr54	45.55±0.78
**39**	Pr09_Fr55	66.8±0.33
**40**	Pr09_Fr56	44.71±0.80
**41**	Pr09_Fr57	29.38±1.20
**42**	Pr09_Fr58	35.07±0.20
**43**	Pr09_Fr59	36.26±0.75

Table S3. List of compounds identified through total and partial NMR-HetCA plots regarding DPPH and collagenase activity. Compounds are colored red or blue according to their contribution or not to the activity, respectively.

**Compounds**	**DPPH**		**Collagenase**	
	Identification from Total NMR-HetCA plots	Identification from Partial NMR-HetCA plots	Identification from Total NMR-HetCA plots	Identification from Partial NMR-HetCA plots
**Pinostrobin (17)**		FR04-07		FR01-04, FR02-05
**Dehydroabietic acid (22)**				FR01-04
**Dehydroabietinol (23)**		FR04-07		FR01-04, FR02-05
**13-*epi*-Torulosal (20)**	Not contributing	FR04-07	Not contributing	FR02-05
**Tectochrysin (15)**	Not contributing	Fr05-08	Not contributing	Fr04-07, Fr08-11
**Isalpinin (16)**				Fr04-07
**pinobanksin-3-(2-methyl)-butyrate (14)**		Fr09-12		Fr09-12, Fr10-13, Fr11-14
**Naringenin (21)**				Fr13-21
**13-*epi*-Cupresic acid (18)**	Not contributing	Fr25-30	Not contributing	Fr14-25, Fr26-31

**Pinocembrin (12)**	Not contributing		Contributing	Fr25-30, Fr26-31
**3-O-methyl galangin (24)**		Fr25-30		Fr25-30, Fr26-31
**Imbricatolic acid (25)**		Fr25-30		Fr25-30, Fr26-31
**Galangin (11)**	Contributing	Fr30-36	Contributing	Fr25-30, Fr26-31
**3-O-Acetyl pinobanksin (10)**	Not contributing	Pr31-37	Not contributing	Fr30-36, Fr31-37
**Chrysin (7)**	Not contributing	Pr36-41	Not contributing	Fr36-41, Fr40-43
**Isocupresic acid (8)**	Not contributing	Pr31-37	Not contributing	Fr36-41, Fr40-43
**Sakuranetin (26)**	Not contributing	Pr45-48	Not contributing	Fr40-43
**Caffeic acid cinnamylester (5)**	Contributing	Pr49-52	Contributing	Fr48-51
**Caffeic acid phenylethylester (1)**	Contributing	Pr49-52	Contributing	Fr49-52, Fr51-54
**3,3-Dimethylallyl caffeate (3)**	Contributing	Pr49-52	Contributing	Fr49-52, Fr51-54
**Isopent-3-enyl caffeate (4)**	Contributing	Pr49-52	Contributing	Fr49-52, Fr51-54
**1-methylpropenyl caffeate (9)**	Contributing	Pr49-52		Fr49-52, Fr51-54
**Caffeic acid benzylester (2)**	Contributing	Pr53-56	Contributing	Fr52-55
**Pinobanksin (6)**	Contributing	Pr53-56		Fr54-57
**8-prenylnaringenin (13)**	Not contributing	Fr06-09, Fr07-10		

**Isogatholal (19)**	Not contributing	Fr08-11, Fr09-12
